# Supplementary material for: A Likelihood Approach for Real-Time Calibration of Stochastic Compartmental Epidemic Models
Source: PLoS Comput Biol. 2017 Jan 17;13(1):e1005257. doi: 10.1371/journal.pcbi.1005257 (PMC5240920; doi:10.1371/journal.pcbi.1005257)
Supplement: S1 File — (TAR.GZ) [file pcbi.1005257.s014.tar.gz › HSPH_Online-SI-Revision/MSS10/n1-extreme/n1-extreme_NEW2_table-8.pdf]

| | | | | | | | | | | | | | | | | | | | | | | | | | | | | | | |
|---|---|---|---|---|---|---|---|---|---|---|---|---|---|---|---|---|---|---|---|---|---|---|---|---|---|---|---|---|---|---|
| 1 | newly 3 weeks spec | 6. | 14.59 | {0., 28.} | 220.167 | 0.704337 | newly 3 weeks cumul | 8. | 27.02 | {1., 46.} | 286.75 | 0.479063 | newly 5 weeks spec | 31. | 27.72 | {0., 69.} | 99.4839 | 0.928818 | newly 5 weeks cumul | 58. | 75.17 | {2., 163.} | 124.397 | 0.65552 | newly 68 weeks cumul | 923. | 575.72 | {2., 920.} | 37.9242 | 0.653148 |
| 2 | newly 3 weeks spec | 47. | 42.65 | {9., 72.} | 48.5745 | 0.276134 | newly 3 weeks cumul | 87. | 84.43 | {30., 139.} | 46.7931 | 0.232117 | newly 5 weeks spec | 79. | 76.4 | {26., 122.} | 39.8228 | 0.236196 | newly 5 weeks cumul | 238. | 222.64 | {79., 372.} | 43.1429 | 0.22892 | newly 64 weeks cumul | 911. | 828.56 | {723., 920.} | 9.54995 | 0.0542548 |
| 3 | newly 3 weeks spec | 17. | 31.83 | {4., 63.} | 133.588 | 0.460557 | newly 3 weeks cumul | 33. | 59.75 | {12., 117.} | 116.515 | 0.337796 | newly 5 weeks spec | 43. | 61.75 | {7., 125.} | 94.0698 | 0.497207 | newly 5 weeks cumul | 103. | 169.54 | {22., 348.} | 107.883 | 0.379596 | newly 66 weeks cumul | 907. | 759. | {531., 906.} | 16.7453 | 0.170014 |
| 4 | newly 3 weeks spec | 11. | 6.62 | {0., 15.} | 89.2727 | 0.928475 | newly 3 weeks cumul | 27. | 16.32 | {2., 36.} | 77.1852 | 0.622001 | newly 5 weeks spec | 51. | 8.74 | {0., 21.} | 87.3333 | 1.53212 | newly 5 weeks cumul | 107. | 32.55 | {2., 74.} | 81.0748 | 0.925606 | newly 65 weeks cumul | 788. | 251.05 | {2., 747.} | 68.5317 | 1.17192 |
| 5 | newly 3 weeks spec | 48. | 4.45 | {1., 9.} | 90.7292 | 1.24693 | newly 3 weeks cumul | 91. | 11.84 | {3., 21.} | 86.989 | 0.991691 | newly 5 weeks spec | 68. | 5.7 | {0., 12.} | 91.6471 | 1.39789 | newly 5 weeks cumul | 214. | 22.76 | {5., 38.} | 89.3645 | 1.10206 | newly 58 weeks cumul | 910. | 290.91 | {25., 486.} | 68.0319 | 0.703493 |
| 6 | newly 3 weeks spec | 34. | 58.41 | {28., 90.} | 81.2059 | 0.258165 | newly 3 weeks cumul | 108. | 127.31 | {67., 192.} | 38.8241 | 0.157532 | newly 5 weeks spec | 27. | 82.11 | {44., 114.} | 206.704 | 0.468817 | newly 5 weeks cumul | 166. | 282.21 | {159., 384.} | 76.5 | 0.239792 | newly 63 weeks cumul | 696. | 781.28 | {641., 883.} | 17.4282 | 0.0716974 |
| 7 | newly 3 weeks spec | 32. | 29.32 | {0., 84.} | 93.6875 | 0.75211 | newly 3 weeks cumul | 67. | 49.6 | {3., 142.} | 81.2537 | 0.629205 | newly 5 weeks spec | 59. | 57.25 | {0., 159.} | 87.1356 | 0.817108 | newly 5 weeks cumul | 173. | 153.27 | {4., 423.} | 85.5087 | 0.661363 | newly 67 weeks cumul | 867. | 698.5 | {6., 931.} | 23.3379 | 0.366266 |
| 8 | newly 3 weeks spec | 33. | 45.79 | {23., 70.} | 53.303 | 0.184782 | newly 3 weeks cumul | 77. | 112.03 | {66., 164.} | 51.961 | 0.170585 | newly 5 weeks spec | 70. | 56.96 | {25., 83.} | 29.3714 | 0.171103 | newly 5 weeks cumul | 203. | 220.86 | {127., 318.} | 30.6305 | 0.129325 | newly 61 weeks cumul | 775. | 633.73 | {411., 806.} | 20.2594 | 0.113735 |
| 9 | newly 3 weeks spec | 10. | 142.5 | {4., 282.} | 1346. | 1.0985 | newly 3 weeks cumul | 20. | 323.14 | {22., 761.} | 1525. | 1.0124 | newly 5 weeks spec | 41. | 75.42 | {0., 156.} | 137.512 | 0.720473 | newly 5 weeks cumul | 80. | 505.2 | {29., 902.} | 550.575 | 0.7928 | newly 68 weeks cumul | 770. | 633.42 | {29., 928.} | 31.4286 | 0.3297 |
| 10 | newly 3 weeks spec | 30. | 16.7 | {4., 31.} | 49.9333 | 0.384914 | newly 3 weeks cumul | 68. | 38.88 | {15., 69.} | 47.3235 | 0.334027 | newly 5 weeks spec | 62. | 25.44 | {5., 51.} | 59.3226 | 0.550263 | newly 5 weeks cumul | 180. | 85.61 | {25., 152.} | 53.4389 | 0.418754 | newly 61 weeks cumul | 869. | 632.87 | {446., 791.} | 27.1795 | 0.199713 |
| 11 | newly 3 weeks spec | 30. | 22.25 | {0., 49.} | 93.7667 | 0.849793 | newly 3 weeks cumul | 41. | 42.96 | {3., 90.} | 97.4634 | 0.536393 | newly 5 weeks spec | 78. | 36.63 | {0., 126.} | 76.7821 | 1.04901 | newly 5 weeks cumul | 165. | 110.71 | {5., 306.} | 83.3515 | 0.716225 | newly 67 weeks cumul | 767. | 548.67 | {5., 884.} | 36.9322 | 0.534546 |
| 12 | newly 3 weeks spec | 6. | 11.83 | {0., 24.} | 189.5 | 0.759514 | newly 3 weeks cumul | 12. | 19.84 | {1., 44.} | 146.333 | 0.514858 | newly 5 weeks spec | 33. | 26.7 | {0., 66.} | 111.333 | 1.18313 | newly 5 weeks cumul | 59. | 67.14 | {2., 133.} | 127.085 | 0.765373 | newly 68 weeks cumul | 724. | 497.06 | {2., 928.} | 49.4751 | 0.809627 |
| 13 | newly 3 weeks spec | 17. | 29.73 | {11., 53.} | 89.7059 | 0.263286 | newly 3 weeks cumul | 47. | 66.83 | {31., 103.} | 56.1489 | 0.188315 | newly 5 weeks spec | 37. | 43.51 | {17., 71.} | 43.2703 | 0.184471 | newly 5 weeks cumul | 107. | 146.6 | {76., 221.} | 52.9346 | 0.18575 | newly 61 weeks cumul | 729. | 703.84 | {563., 810.} | 10.2112 | 0.051915 |
| 14 | newly 3 weeks spec | 25. | 67.84 | {30., 113.} | 176.72 | 0.413117 | newly 3 weeks cumul | 67. | 137.34 | {70., 217.} | 108.896 | 0.294853 | newly 5 weeks spec | 83. | 103.03 | {67., 140.} | 37.9639 | 0.205589 | newly 5 weeks cumul | 206. | 328.87 | {180., 470.} | 67.4515 | 0.220405 | newly 65 weeks cumul | 782. | 821.82 | {747., 911.} | 12.2864 | 0.0646238 |
| 15 | newly 3 weeks spec | 26. | 34.77 | {18., 57.} | 49.6538 | 0.179332 | newly 3 weeks cumul | 51. | 80.55 | {43., 123.} | 65.5098 | 0.211592 | newly 5 weeks spec | 70. | 49.92 | {26., 71.} | 31.7429 | 0.200846 | newly 5 weeks cumul | 147. | 174.04 | {101., 255.} | 35.1973 | 0.143573 | newly 60 weeks cumul | 692. | 727.65 | {631., 829.} | 12.2962 | 0.0624639 |
| 16 | newly 3 weeks spec | 24. | 9.81 | {0., 20.} | 84.375 | 1.00686 | newly 3 weeks cumul | 40. | 17.96 | {1., 34.} | 80.3 | 0.757349 | newly 5 weeks spec | 42. | 18.32 | {0., 48.} | 82.2381 | 1.09987 | newly 5 weeks cumul | 120. | 50.84 | {2., 105.} | 80.3667 | 0.857673 | newly 67 weeks cumul | 755. | 554.2 | {2., 901.} | 38.4662 | 0.647063 |
| 17 | newly 3 weeks spec | 26. | 9.54 | {1., 21.} | 65.9231 | 0.719652 | newly 3 weeks cumul | 47. | 20.53 | {3., 43.} | 60.7021 | 0.542324 | newly 5 weeks spec | 50. | 17.02 | {0., 35.} | 69.36 | 0.87708 | newly 5 weeks cumul | 121. | 50.67 | {4., 111.} | 63.4793 | 0.621499 | newly 64 weeks cumul | 819. | 592.65 | {4., 856.} | 29.6813 | 0.452152 |
| 18 | newly 3 weeks spec | 13. | 42.67 | {2., 100.} | 261.462 | 0.559799 | newly 3 weeks cumul | 23. | 82.62 | {12., 167.} | 276.87 | 0.49914 | newly 5 weeks spec | 42. | 64.26 | {1., 150.} | 106.381 | 0.575142 | newly 5 weeks cumul | 99. | 204.55 | {23., 439.} | 143.323 | 0.428093 | newly 67 weeks cumul | 912. | 616.79 | {67., 907.} | 32.8235 | 0.337727 |
| 19 | newly 3 weeks spec | 14. | 43.94 | {0., 127.} | 286. | 0.803016 | newly 3 weeks cumul | 34. | 77.42 | {3., 196.} | 196.824 | 0.587864 | newly 5 weeks spec | 39. | 73.68 | {0., 184.} | 163.128 | 0.84576 | newly 5 weeks cumul | 99. | 216.81 | {3., 568.} | 187.667 | 0.6688 | newly 68 weeks cumul | 880. | 677.51 | {3., 933.} | 26.233 | 0.434857 |
| 20 | newly 3 weeks spec | 50. | 48.98 | {28., 74.} | 28.16 | 0.142034 | newly 3 weeks cumul | 121. | 119.96 | {74., 174.} | 26.1818 | 0.124057 | newly 5 weeks spec | 81. | 64.26 | {37., 90.} | 25.7284 | 0.148655 | newly 5 weeks cumul | 273. | 243.64 | {164., 338.} | 22.2564 | 0.116179 | newly 61 weeks cumul | 893. | 692.98 | {471., 812.} | 22.3987 | 0.120595 |
| 21 | newly 3 weeks spec | 10. | 37.92 | {17., 65.} | 285.6 | 0.565366 | newly 3 weeks cumul | 39. | 78.74 | {35., 120.} | 111.077 | 0.305715 | newly 5 weeks spec | 27. | 62.53 | {25., 93.} | 141.148 | 0.386824 | newly 5 weeks cumul | 84. | 189.33 | {79., 297.} | 133.583 | 0.35422 | newly 64 weeks cumul | 711. | 810.19 | {701., 898.} | 19.5373 | 0.0871698 |
| 22 | newly 3 weeks spec | 50. | 36.33 | {7., 68.} | 49.98 | 0.41045 | newly 3 weeks cumul | 92. | 73.76 | {18., 135.} | 45.5 | 0.296006 | newly 5 weeks spec | 80. | 63.61 | {7., 116.} | 45.4875 | 0.394876 | newly 5 weeks cumul | 222. | 187.22 | {43., 337.} | 45.7658 | 0.311985 | newly 65 weeks cumul | 825. | 709.7 | {412., 902.} | 18.9891 | 0.188179 |
| 23 | newly 3 weeks spec | 21. | 9.4 | {0., 23.} | 72.4762 | 0.831037 | newly 3 weeks cumul | 38. | 17.96 | {2., 41.} | 69.2632 | 0.647524 | newly 5 weeks spec | 50. | 20.84 | {0., 56.} | 77.92 | 1.0709 | newly 5 weeks cumul | 115. | 52.67 | {2., 127.} | 74.3565 | 0.774668 | newly 67 weeks cumul | 744. | 549.03 | {3., 883.} | 37.1196 | 0.572075 |
| 24 | newly 3 weeks spec | 11. | 10.47 | {0., 23.} | 113.545 | 0.897911 | newly 3 weeks cumul | 16. | 17.28 | {1., 39.} | 105.75 | 0.57885 | newly 5 weeks spec | 41. | 25.68 | {0., 73.} | 99.8537 | 1.29698 | newly 5 weeks cumul | 85. | 60.81 | {1., 166.} | 99.8941 | 0.890841 | newly 68 weeks cumul | 886. | 453.68 | {2., 915.} | 50.0519 | 0.963967 |
| 25 | newly 3 weeks spec | 21. | 9.83 | {0., 21.} | 71.2857 | 0.715243 | newly 3 weeks cumul | 34. | 20.33 | {3., 35.} | 64.2059 | 0.451449 | newly 5 weeks spec | 31. | 18.31 | {0., 40.} | 66.0968 | 0.668166 | newly 5 weeks cumul | 93. | 53.19 | {7., 96.} | 63.2581 | 0.504923 | newly 65 weeks cumul | 758. | 574.83 | {9., 839.} | 30.7533 | 0.409088 |
| 26 | newly 3 weeks spec | 12. | 12.24 | {1., 27.} | 85.8333 | 0.579885 | newly 3 weeks cumul | 15. | 22.88 | {3., 48.} | 102.533 | 0.363192 | newly 5 weeks spec | 48. | 29.72 | {0., 69.} | 69. | 0.834237 | newly 5 weeks cumul | 84. | 72.23 | {5., 173.} | 75.4167 | 0.523932 | newly 67 weeks cumul | 923. | 639.47 | {9., 915.} | 30.9805 | 0.444159 |
| 27 | newly 3 weeks spec | 38. | 25.36 | {6., 44.} | 51.4211 | 0.378275 | newly 3 weeks cumul | 98. | 61.92 | {24., 110.} | 49.9796 | 0.337736 | newly 5 weeks spec | 56. | 31.52 | {4., 61.} | 50.2857 | 0.47826 | newly 5 weeks cumul | 192. | 123.39 | {38., 205.} | 45.8177 | 0.329804 | newly 62 weeks cumul | 795. | 534.06 | {104., 728.} | 32.8906 | 0.283846 |
| 28 | newly 3 weeks spec | 39. | 102.7 | {40., 164.} | 167.846 | 0.406756 | newly 3 weeks cumul | 74. | 194.46 | {71., 311.} | 167.784 | 0.386327 | newly 5 weeks spec | 88. | 125.11 | {72., 156.} | 52.0568 | 0.212652 | newly 5 weeks cumul | 201. | 444.98 | {225., 627.} | 126.229 | 0.345885 | newly 66 weeks cumul | 916. | 831.75 | {731., 918.} | 9.40939 | 0.0638808 |
| 29 | newly 3 weeks spec | 8. | 12.58 | {4., 24.} | 84.75 | 0.295496 | newly 3 weeks cumul | 18. | 27.29 | {10., 46.} | 71.9444 | 0.234852 | newly 5 weeks spec | 61. | 23.24 | {2., 48.} | 64.8525 | 0.656656 | newly 5 weeks cumul | 116. | 68.16 | {16., 126.} | 51.2759 | 0.368293 | newly 63 weeks cumul | 930. | 671.91 | {333., 851.} | 27.7516 | 0.253096 |
| 30 | newly 3 weeks spec | 51. | 57.28 | {37., 76.} | 24.4314 | 0.0977625 | newly 3 weeks cumul | 155. | 161.42 | {117., 204.} | 18.2839 | 0.0778098 | newly 5 weeks spec | 44. | 54.47 | {35., 72.} | 31.2955 | 0.11799 | newly 5 weeks cumul | 251. | 273.84 | {199., 341.} | 18.1195 | 0.0741758 | newly 58 weeks cumul | 605. | 528.27 | {439., 608.} | 14.4182 | 0.0700253 |
| 31 | newly 3 weeks spec | 24. | 12.33 | {0., 30.} | 81.875 | 0.918197 | newly 3 weeks cumul | 52. | 23.58 | {2., 47.} | 76.4615 | 0.715472 | newly 5 weeks spec | 60. | 29.07 | {0., 81.} | 72.6833 | 1.00612 | newly 5 weeks cumul | 138. | 72.45 | {3., 167.} | 74.7029 | 0.734917 | newly 67 weeks cumul | 874. | 601.23 | {3., 926.} | 33.6144 | 0.610752 |
| 32 | newly 3 weeks spec | 21. | 78.24 | {14., 161.} | 287.714 | 0.590719 | newly 3 weeks cumul | 42. | 134.84 | {29., 274.} | 235.619 | 0.487058 | newly 5 weeks spec | 41. | 118.09 | {54., 176.} | 198.707 | 0.526293 | newly 5 weeks cumul | 113. | 362.46 | {121., 607.} | 231.735 | 0.51245 | newly 67 weeks cumul | 845. | 807.54 | {562., 939.} | 13.2781 | 0.116811 |
| 33 | newly 3 weeks spec | 14. | 1.8 | {0., 4.} | 87.1429 | 1.25468 | newly 3 weeks cumul | 41. | 4.97 | {1., 9.} | 87.878 | 1.06301 | newly 5 weeks spec | 35. | 2.43 | {0., 6.} | 93.0571 | 1.66586 | newly 5 weeks cumul | 96. | 9.57 | {2., 18.} | 90.0313 | 1.18791 | newly 63 weeks cumul | 860. | 161.89 | {2., 474.} | 81.1756 | 1.37127 |
| 34 | newly 3 weeks spec | 20. | 50.41 | {4., 97.} | 195.75 | 0.534458 | newly 3 weeks cumul | 48. | 93.38 | {10., 177.} | 135.417 | 0.389686 | newly 5 weeks spec | 70. | 67.75 | {3., 130.} | 60.7 | 0.515924 | newly 5 weeks cumul | 151. | 222.91 | {22., 467.} | 98.7086 | 0.423168 | newly 66 weeks cumul | 845. | 707.44 | {262., 917.} | 21.3893 | 0.227195 |
| 35 | newly 3 weeks spec | 18. | 9.16 | {0., 11.} | 103.444 | 1.07541 | newly 3 weeks cumul | 50. | 19.61 | {1., 20.} | 98.94 | 0.959679 | newly 5 weeks spec | 58. | 12.1 | {0., 28.} | 89.0345 | 1.47893 | newly 5 weeks cumul | 143. | 42.02 | {2., 66.} | 93.0769 | 1.10049 | newly 67 weeks cumul | 834. | 440.58 | {3., 848.} | 48.8777 | 0.804878 |
| 36 | newly 3 weeks spec | 24. | 21.69 | {5., 44.} | 48.625 | 0.289285 | newly 3 weeks cumul | 59. | 46.88 | {17., 90.} | 42.3729 | 0.253106 | newly 5 weeks spec | 53. | 38.26 | {9., 69.} | 45.1321 | 0.319982 | newly 5 weeks cumul | 146. | 114.92 | {33., 210.} | 42.2055 | 0.264881 | newly 64 weeks cumul | 848. | 692.59 | {463., 845.} | 19.0035 | 0.14639 |
| 37 | newly 3 weeks spec | 45. | 15.18 | {2., 30.} | 66.3111 | 0.664601 | newly 3 weeks cumul | 72. | 36.96 | {10., 62.} | 51.6389 | 0.401453 | newly 5 weeks spec | 59. | 22.19 | {2., 41.} | 62.3898 | 0.644065 | newly 5 weeks cumul | 179. | 76.95 | {17., 140.} | 57.0112 | 0.483065 | newly 59 weeks cumul | 848. | 524.93 | {137., 694.} | 38.0979 | 0.349347 |
| 38 | newly 3 weeks spec | 27. | 48.5 | {18., 82.} | 92. | 0.266743 | newly 3 weeks cumul | 70. | 93.71 | {42., 159.} | 54.3286 | 0.195792 | newly 5 weeks spec | 62. | 83.76 | {44., 121.} | 48.5806 | 0.176757 | newly 5 weeks cumul | 186. | 245.06 | {115., 390.} | 48.4624 | 0.177839 | newly 64 weeks cumul | 878. | 865.13 | {786., 923.} | 5.11276 | 0.0228776 |
| 39 | newly 3 weeks spec | 9. | 5.51 | {0., 12.} | 86.5556 | 0.872154 | newly 3 weeks cumul | 17. | 11.79 | {1., 29.} | 81.2353 | 0.591281 | newly 5 weeks spec | 28. | 11.66 | {0., 30.} | 86.0714 | 1.25103 | newly 5 weeks cumul | 66. | 31.72 | {2., 74.} | 81.2121 | 0.844438 | newly 67 weeks cumul | 893. | 374.13 | {2., 865.} | 58.5342 | 1.04569 |
| 40 | newly 3 weeks spec | 58. | 69.19 | {28., 117.} | 46.6724 | 0.216793 | newly 3 weeks cumul | 138. | 136.71 | {59., 226.} | 37.6449 | 0.193237 | newly 5 weeks spec | 45. | 104.14 | {56., 142.} | 134.889 | 0.377926 | newly 5 weeks cumul | 245. | 332.86 | {159., 496.} | 49.9265 | 0.188851 | newly 65 weeks cumul | 775. | 836.69 | {727., 916.} | 12.6955 | 0.0665846 |
| 41 | newly 3 weeks spec | 31. | 27.61 | {1., 73.} | 62.6774 | 0.498567 | newly 3 weeks cumul | 62. | 77.64 | {18., 161.} | 71.8065 | 0.310226 | newly 5 weeks spec | 37. | 25.33 | {0., 51.} | 54.4595 | 0.655902 | newly 5 weeks cumul | 130. | 129.18 | {27., 261.} | 54.6154 | 0.318726 | newly 60 weeks cumul | 655. | 373.07 | {35., 612.} | 43.058 | 0.4478 |
| 42 | newly 3 weeks spec | 20. | 8.3 | {0., 21.} | 85.7 | 1.07321 | newly 3 weeks cumul | 35. | 14.83 | {2., 35.} | 77.4 | 0.735163 | newly 5 weeks spec | 52. | 21.57 | {0., 67.} | 83.0192 | 1.15627 | newly 5 weeks cumul | 108. | 50.12 | {2., 139.} | 80.9815 | 0.867412 | newly 68 weeks cumul | 918. | 526.56 | {2., 920.} | 42.8627 | 0.765295 |
| 43 | newly 3 weeks spec | 9. | 8.34 | {0., 13.} | 104.444 | 0.842646 | newly 3 weeks cumul | 17. | 16.21 | {1., 25.} | 93.7059 | 0.536325 | newly 5 weeks spec | 64. | 17.14 | {0., 43.} | 84.4688 | 1.33814 | newly 5 weeks cumul | 106. | 47.08 | {2., 110.} | 85.434 | 0.893388 | newly 67 weeks cumul | 902. | 515.37 | {2., 896.} | 43.2605 | 0.741171 |
| 44 | newly 3 weeks spec | 40. | 23.43 | {4., 46.} | 57.175 | 0.47728 | newly 3 weeks cumul | 67. | 44.8 | {10., 92.} | 54.5373 | 0.380689 | newly 5 weeks spec | 68. | 49.18 | {9., 103.} | 52.3529 | 0.42929 | newly 5 weeks cumul | 179. | 129.47 | {23., 261.} | 54.6536 | 0.380142 | newly 65 weeks cumul | 873. | 794.08 | {645., 927.} | 12.4147 | 0.109011 |
| 45 | newly 3 weeks spec | 11. | 30.39 | {0., 79.} | 251. | 0.731306 | newly 3 weeks cumul | 17. | 55.37 | {3., 131.} | 283.706 | 0.533386 | newly 5 weeks spec | 50. | 51.61 | {0., 167.} | 107.98 | 1.02842 | newly 5 weeks cumul | 90. | 152.23 | {3., 453.} | 157.633 | 0.701147 | newly 68 weeks cumul | 926. | 573.24 | {3., 932.} | 38.4212 | 0.630387 |
| 46 | newly 3 weeks spec | 19. | 8.06 | {0., 22.} | 83.0526 | 1.0568 | newly 3 weeks cumul | 34. | 18.32 | {2., 41.} | 79.2353 | 0.683914 | newly 5 weeks spec | 55. | 11.94 | {0., 30.} | 82.9818 | 1.34428 | newly 5 weeks cumul | 129. | 39.57 | {3., 92.} | 80.1628 | 0.930691 | newly 65 weeks cumul | 889. | 316.68 | {3., 767.} | 64.5309 | 1.0117 |
| 47 | newly 3 weeks spec | 19. | 33.17 | {3., 76.} | 119.632 | 0.451246 | newly 3 weeks cumul | 29. | 66.11 | {9., 137.} | 157.069 | 0.416052 | newly 5 weeks spec | 39. | 57.66 | {0., 118.} | 100.308 | 0.622974 | newly 5 weeks cumul | 94. | 168.68 | {13., 352.} | 119.149 | 0.4285 | newly 66 weeks cumul | 840. | 637.44 | {14., 896.} | 27.831 | 0.352889 |
| 48 | newly 3 weeks spec | 17. | 98.99 | {38., 172.} | 482.294 | 0.704385 | newly 3 weeks cumul | 48. | 194.93 | {94., 336.} | 306.104 | 0.551441 | newly 5 weeks spec | 41. | 121.41 | {79., 152.} | 197.976 | 0.466567 | newly 5 weeks cumul | 126. | 440.33 | {241., 634.} | 249.69 | 0.516157 | newly 66 weeks cumul | 757. | 844.63 | {748., 913.} | 13.5443 | 0.0551173 |
| 49 | newly 3 weeks spec | 30. | 18.87 | {0., 54.} | 86.9667 | 0.912028 | newly 3 weeks cumul | 64. | 39.6 | {2., 79.} | 91.5 | 0.754512 | newly 5 weeks spec | 69. | 43.15 | {0., 140.} | 80.6812 | 1.0214 | newly 5 weeks cumul | 166. | 113.62 | {3., 334.} | 85.0241 | 0.770838 | newly 68 weeks cumul | 950. | 612.14 | {3., 925.} | 35.5642 | 0.567421 |
| 50 | newly 3 weeks spec | 39. | 107.01 | {32., 172.} | 187.462 | 0.518291 | newly 3 weeks cumul | 92. | 254.98 | {100., 417.} | 187.304 | 0.457201 | newly 5 weeks spec | 56. | 79.26 | {6., 132.} | 73. | 0.461157 | newly 5 weeks cumul | 194. | 434. | {170., 628.} | 136.155 | 0.411246 | newly 66 weeks cumul | 717. | 611.94 | {248., 887.} | 28.1199 | 0.224683 |
